# Linkage mapping of a complex trait in the New York population of the GAW14 simulated dataset: a multivariate phenotype approach

**DOI:** 10.1186/1471-2156-6-S1-S19

**Published:** 2005-12-30

**Authors:** Saurabh Ghosh, Samsiddhi Bhattacharjee, Gourab Basu, Sandip Pal, Partha P Majumder

**Affiliations:** 1 Human Genetics Unit, Indian Statistical Institiute, 203 B.T. Road, Kolkata 700 108, India

## Abstract

Multivariate phenotypes underlie complex traits. Thus, instead of using the end-point trait, it may be statistically more powerful to use a multivariate phenotype correlated to the end-point trait for detecting linkage. In this study, we develop a reverse regression method to analyze linkage of Kofendrerd Personality Disorder affection status in the New York population of the Genetic Analysis Workshop 14 (GAW14) simulated dataset. When we used the multivariate phenotype, we obtained significant evidence of linkage near four of the six putative loci in at least 25% of the replicates. On the other hand, the linkage analysis based on Kofendrerd Personality Disorder status as a phenotype produced significant findings only near two of the loci and in a smaller proportion of replicates.

## Background

A complex trait is usually a function of a multivariate phenotype comprising correlated quantitative variables. Since end-point traits are usually binary in nature (affected/unaffected) and hence contain minimal information on variation within trait genotypes, it may be statistically more powerful to use a correlated multivariate phenotype for identifying genes for the complex trait. Mapping a multivariate phenotype traditionally uses some function of quantitative values of sib-pairs or other sets of relatives as a response variable and marker identity-by-descent (IBD) scores as explanatory variables [1-3]. In these analyses, linkage inferences depend strongly on the assumed probability distributions of the quantitative variables, particularly for likelihood-based approaches such as variance components [3,4]. We propose a linear regression formulation in which the response and explanatory variables are interchanged, such as that used by Sham et al. [5]. Analyses do not require modeling the covariance structure of the multivariate phenotype vector [2-4] or any data reduction technique, such as principal components [6]. In this study, we use the proposed method for performing a genome-wide scan of a multivariate phenotype vector correlated with Kofendred Personality Disorder (KPD) in the New York population of the simulated dataset of GAW14.

## Methods

### Data description

For our analysis, we considered data on the KPD status (affected or unaffected), twelve associated binary phenotypes, and genome-wide information separately on 416 microsatellite marker loci and 917 single nucleotide polymorphisms (SNPs) with average intermarker distances of 7.5 cM and 3 cM, respectively, distributed over 10 autosomal chromosomes for the New York population. Our method utilizes phenotype and marker data on 50 independent sibships of sizes varying from 2 to 9 and their parental genotypes for IBD computations. We analyzed data on all 100 available replicates.

### Constructing the multivariate phenotype

Suppose *y*_*ijk *_denote the phenotypic value of the *i*^th ^trait for the *j*^th ^sib in the *k*^th^ sibship, *i *= 1, 2, 3, 4, 5; *j *= 1, 2, ..., *n*_*k*_; *k *= 1, 2, ..., 50. The twelve phenotypes relate to personality traits and therefore may be associated with the end-point trait, the affectation status of KPD. Thus, instead of using the KPD status as a phenotype for linkage analysis, it may be statistically more powerful to use a multivariate phenotype comprising some of these personality traits, which are highly correlated to the disease status. In order to select a subset of the twelve traits, which may be used as a surrogate for the end-point trait, we performed a logistic regression of the KPD disease status on the twelve binary phenotypes. To ensure the independence of our observations, the regression was based on the 100 parents of the 50 sibships.

The logistic model used was:



where *z*_*jk *_is the affectation status of KPD of the *j*^th ^parent of the *k*^th ^sibship; *δ *= 0 or 1 according to whether an individual is affected with KPD or not and *x*_*ijk *_is the phenotypic value of the *i*^th ^trait of the *j*^th ^parent of the *k*^th ^sibship. The test for association between the *i*^th ^(i = 1, 2, ..., 12) personality trait with KPD is equivalent to testing *a*_*i *_= 0 versus *a*_*i *_≠ 0. We used a level of 0.005 for testing each *a*_*i *_in the 12 tests. We obtained five of the phenotypes to be significantly correlated to the end-point trait (details are provided in the "Results" section). Thus, the multivariate phenotype we used for our linkage analysis comprises five binary personality traits.

### The reverse regression procedure

Sham et al. [5] proposed a regression method that interchanges the phenotype and the marker IBD score variables. We adapted their method for the following linear regression model:



where  is the estimated marker IBD score of the first and *j*^th ^sibs of the *k*^th ^sibship, *j *= 1, 2, ..., *n*_*k*_; *k *= 1, 2, ..., 50; *e*_*jk *_values are random environmental errors assumed to have mean 0 and equal variances. We note here that an advantage of using IBD scores instead of the squared sib-pair trait differences as the response variable is that in a sibship of size *n*_*k*_, the marker IBD scores *π*_1*k*,2*k*_, *π*_1*k*,3*k*_, ..., *π*_1*k*_,*n*_*k *_are independent, but the squared differences in trait values for these sib-pairs are not independent. Thus, for a sibship of size *n*_*k*_, we have *n*_*k *_- 1 independent. We wish to point out here that our method is not related to parity (i.e., birth order). While analyzing data, we suggest that the sib assigned "1" be chosen at random from the sibship. When we computed multipoint IBD scores, the conversion of recombination distances to physical distances (in cM) on chromosomes was based on the Haldane map function [7].

We define our test for linkage between the locus controlling KPD and the marker locus to be equivalent to testing *H*_0 _: β_1 _= β_2 _= β_3 _= β_4 _= β_5 _= 0 versus *H*_1_: β_1 _< 0 *U *β_2 _< 0 *U *β_3 _< 0 *U *β_4 _< 0 *U *β_5 _< 0. In other words, under no linkage between the two loci, the estimated marker IBD score will not be correlated to the squared difference in sib-pair trait values. On the other hand, if the two loci are linked, the estimated marker IBD score will not be correlated to the squared difference in sib-pair values for at least one of the correlated traits [1].

The test statistic used is



The above statistic is equivalent to the usual likelihood ratio test (LRT) for normally distributed errors. Under the assumption of normality, the test statistic is distributed asymptotically as a mixture of chi-square distributions. It is very unlikely in practice for the errors to be distributed as normal. Thus, instead of making any assumptions on the distribution of the errors, we use Monte Carlo simulations to obtain the empirical *p*-values for the observed value of the test statistic. We generate marker IBD scores at random using the marginal distribution of IBD scores (based on a multiallelic modification of Table V in Haseman and Elston [1] and marker allele frequencies as provided in the dataset) and assign them to the different sib-pairs in the regression analysis. The squared differences in the phenotypic values of the sib-pairs are conserved and the regression is performed to generate values of the test statistic under the null hypothesis of no linkage.

Because our aim is to show that using the multivariate phenotype vector for the linkage scan is statistically more powerful than using the end-point trait (KPD status), we also perform the reverse regression analysis using only the KPD status. The regression procedure is identical to the one described above with the test for linkage based on only one parameter, i.e., the regression coefficient associated with the KPD status variable.

## Results

Based on the logistic regression, five phenotypes: fear/discomfort with strangers, dislike of jokes told face to face, obsession with entertainers, humor impairment, and uncommunicative, contentless speech patterns were found to be significantly correlated to KPD status. As mentioned earlier, we performed two linkage analyses: one based on a multivariate phenotype vector comprising these five traits and the other based on only the KPD status as a phenotype. We used the statistical package MERLIN 0.10.2 [8] for multipoint IBD computations. The reverse regression method described above was performed at the marker/SNP positions. The test for linkage had level 0.001 (for each marker) and the null distribution of the test statistic was determined using 1,000 Monte-Carlo simulations. Since the "answers" were available to us, we considered a linkage peak to be true positive if it is within 10 cM from the true position of the putative locus. The results are provided in Table 1 for the end-point trait and in Table 2 for the multivariate phenotype in terms of the proportion of replicates where significant linkage peaks were obtained along with the markers within 10 cM of those peaks.

When we used the multivariate phenotype, the linkage analyses based on the 416 microsatellite markers yielded significant peaks on 4 chromosomes: D01S0023 on chromosome 1, D03S0127 on chromosome 3, D05S0173 on chromosome 5, and D09S0347 on chromosome 9. The linkage analyses using the 917 SNP markers yielded significant peaks around the same regions as the peaks corresponding to the microsatellite markers: C01R0051 on chromosome 1, C03R0281 on chromosome 3, C05R0381 on chromosome 5, and C09R0763 on chromosome 9. When we used the end-point KPD status as our phenotype, we obtained significant peaks only at D03S0127 and C03R0281 on chromosome 3; and D05S0173 and C05R0381 on chromosome 5 for microsatellite markers and SNPs, respectively. It is clear from the tables that not only did the multivariate phenotype approach produce significant linkage findings at more locations, but also the proportions of replicates in which we obtained the significant findings for both microsatellite markers and SNPs were much lower when only the KPD status was used.

Based on the multivariate phenotype, we have been able to detect linkage in at least 25% of the replicates for both microsatellite markers and SNP markers on four chromosomes (1, 3, 5, and 9) very close to the putative trait loci. The proportion of replicates in which we obtained significant linkage findings for the SNPs appears to be marginally lower than that for the microsatellite markers. This can be explained by the fact that since the SNPs are less polymorphic compared with microsatellite markers, the information content at the same marker density is higher with microsatellite markers, leading to more efficient estimation of marker IBD scores. Moreover, we used the same level of significance in our tests of linkage for both microsatellite as well as SNP markers. Since the SNPs are at a much higher density, at the same level of single-marker significance, the genome-wide significance level based on SNPs is higher than that for the microsatellite markers.

## Conclusion

Our proposed reverse regression method was able to detect linkage near four of the six putative loci controlling KPD in multiple replicates. We found that our linkage analyses based on the multivariate phenotype comprising five binary traits correlated with KPD was more powerful than those based on only the affectation status of KPD as the phenotype. Thus, using a multivariate phenotype vector comprising traits correlated with the end-point trait may be a prudent strategy for linkage mapping of a complex trait.

While it is important to compare the power of our method with those of existing methodologies, the structure of the dataset did not permit a valid statistical comparison with most existing methods. The variance components methods like those implemented in MERLIN, GENEHUNTER, SEGPATH, and ACT assume multivariate normality of trait values within pedigrees and are designed for quantitative traits. However, all the personality traits in the dataset were binary in nature and assumption of normality for these traits would not be proper. The package SOLAR has an option of using a threshold model for binary traits [9], but like MERLIN and GENEHUNTER, allows for single traits only. Thus, it was difficult to compare our method with other multivariate methods. While we showed that using the multivariate phenotype yields more power than using only KPD status based on the reverse regression strategy, it is of interest to explore whether our multivariate method is more powerful than standard univariate analyses on KPD status implemented in LINKAGE or GENEHUNTER. However, a direct comparison with LINKAGE is difficult because it is parametric in nature and would yield LOD scores as the linkage statistic. Since our method is completely model-free, it is not possible to compute LOD equivalents from our statistic. On the other hand, because our analyses involved affected and unaffected individuals, it would not be proper to compare with an analysis involving only affected individuals as implemented in model-free analyses of GENEHUNTER. We may have missed out on valid comparisons with some other existing methodologies and are currently exploring those possibilities.

The overall level of significance would most likely be a function of the level of significance used in the first stage of our analysis in which we are selecting a subset of phenotypes that are significantly associated with the end-point trait. The nature of dependence of the two stages is quite complex and it is difficult to obtain exact adjustments of the *p*-values in the linkage scan after accounting for the *p*-values in the first stage. Extensive simulations to examine this issue are being conducted.

## Abbreviations

GAW14: Genetic Analysis Workshop 14

IBD: Identity by descent

KPD: Kofendrerd Personality Disorder

LRT: Likelihood ratio test

SNP: Single-nucleotide polymorphism

## Authors' contributions

SG proposed and worked on the methodology. SB optimized the linkage statistics and wrote the computer codes. GB and SP managed the data and implemented the software packages/computer programs for IBD computations, logistic regression, and empirical power computations. PPM coordinated the analysis and participated in writing the manuscript.

## Figures and Tables

**Table 1:** Significant linkage peaks and microsatellite markers/SNPs within 10 cM of the peaks based on the KPD status

Chr	Marker Name	Position (in cM)	PR^a^	SNP Name	Position (in cM)	PR
3	D03S0126	306.073	0.17	C03R0279	297.181	0.15
	D03S0127	313.922	0.22*	C03R0280	300.112	0.18
				C03R0281	303.303	0.19*
5	D05S0172	0.0	0.14	C05R0380	0.0	0.15
	D05S0173	7.84	0.18*	C05R0381	2.271	0.17
	D05S0174	15.576	0.13	C05R0382	5.307	0.15
				C05R0383	8.517	0.14

^a^PR: Proportion of replicates yielding significant results, *indicates peaks

**Table 2:** Significant linkage peaks and microsatellite markers/SNPs within 10 cM of the peaks based on the multivariate phenotype vector.

Chr	Marker Name	Position (in cM)	PR^a^	SNP Name	Position (in cM)	PR
1	D01S0022	164.328	0.37	C01R0049	162.594	0.33
	D01S0023	173.616	0.46*	C01R0050	166.784	0.35
	D01S0024	181.157	0.38	C01R0051	170.013	0.42*
				C01R0052	173.193	0.40
				C01R0053	175.727	0.34
				C01R0054	179.314	0.28
3	D03S0126	306.073	0.47	C03R0277	297.181	0.29
	D03S0127	313.922	0.51*	C03R0278	300.112	0.33
				C03R0279	303.303	0.39
				C03R0280	305.768	0.42
				C03R0281	308.234	0.46*
5	D05S0172	0.0	0.27	C05R0378	0.0	0.25
	D05S0173	7.84	0.35*	C05R0379	2.271	0.27
	D05S0174	15.576	0.27	C05R0380	5.307	0.28
				C05R0381	8.517	0.32*
				C05R0382	11.454	0.31
				C05R0383	14.74	0.27
				C05R0384	17.249	0.26
9	D09S0347	0.0	0.41*	C09R0763	0.00	0.40*
	D09S0348	8.105	0.34	C09R0764	2.846	0.37
				C09R0765	5.672	0.32
				C09R0766	9.233	0.30
				C09R0767	11.402	0.27

^a^PR: Proportion of replicates yielding significant results, *indicates peaks
